# The Functions of SARS-CoV-2 Receptors in Diabetes-Related Severe COVID-19

**DOI:** 10.3390/ijms25179635

**Published:** 2024-09-05

**Authors:** Adam Drzymała

**Affiliations:** Department of Clinical Biochemistry and Laboratory Diagnostics, Institute of Medical Sciences, University of Opole, Oleska 48, 45-052 Opole, Poland; adam.drzymala@uni.opole.pl

**Keywords:** SARS-CoV-2, diabetes mellitus type 2, ACE2, CD147, GRP78, CD4, TfR, NRP2, integrins, vimentin, sialic acid

## Abstract

Angiotensin-converting enzyme 2 (ACE2) is considered a severe acute respiratory syndrome coronavirus 2 (SARS-CoV-2) receptor of high importance, but due to its non-ubiquitous expression, studies of other proteins that may participate in virus internalisation have been undertaken. To date, many alternative receptors have been discovered. Their functioning may provide an explanation for some of the events observed in severe COVID-19 that cannot be directly explained by the model in which ACE2 constitutes the central point of infection. Diabetes mellitus type 2 (T2D) can induce severe COVID-19 development. Although many mechanisms associated with ACE2 can lead to increased SARS-CoV-2 virulence in diabetes, proteins such as basigin (CD147), glucose-regulated protein 78 kDa (GRP78), cluster of differentiation 4 (CD4), transferrin receptor (TfR), integrins α_5_β_1_/α_v_β_3_, or ACE2 co-receptors neuropilin 2 (NRP2), vimentin, and even syalilated gangliosides may also be responsible for worsening the COVID-19 course. On the other hand, some others may play protective roles. Understanding how diabetes-associated mechanisms can induce severe COVID-19 via modification of virus receptor functioning needs further extensive studies.

## 1. Introduction

Since the outbreak of the COVID-19 pandemic in 2020, many reports have discussed the causes of the disease’s severity. The observations that certain co-morbidities may underline it have prompted searches for a link between these diseases and COVID-19. One such disease is diabetes mellitus type 2. Clinical data indicate that the risk of severe COVID-19 development among patients with T2D is estimated at OR = 1.8 [1]. In the group of increased risk are also patients over 60 years old and those with hypertension [2] or obesity [3]. The mechanism of infection embraces ACE2 as the main SARS-CoV-2 receptor. However, due to the possibility of multiple organ dysfunction syndrome (MODS) development and a non-ubiquitous expression profile of the protein, combined with the presence of modifications affecting ACE2-RBD (receptor-binding domain of SARS-CoV-2 spike protein) interactions [4,5,6], attempts to find other potential receptors that could justify the events observed in the severe COVID-19 course have been undertaken. Moreover, various reports indicate the reduced effect of RBD-based COVID-19 vaccines in diabetic patients. Ali et al. revealed that the abundance of neutralising antibodies was lower by 4.42% in T2D patients after inoculation with BNT162b2 [7], whereas the difference in the study by Tawinprai et al. equalled even to 55% (AZD1222) [8]. Uncontrolled hyperglycaemia may also affect the level of neutralising antibodies. The differences between groups of diabetic patients with various haemoglobin glycation levels (HbA1c 48 and 65 mmol/L, respectively) were clearly visible after 21 days, as well as 52 days after the second vaccination. Moreover, introduction of appropriate glycaemic control after inoculation increased the immune response on day 52 [9]. The possibility of severe COVID-19 occurrence after vaccination varied within studies from OR = 1.03 up to 2.41 in the comparison to the control group of patients due to high heterogeneity [10]. On the other hand, some reports indicate even higher effectiveness of vaccines in diabetic patients [11], but overall comparison tends to lead to the conclusion that the reaction of diabetic patients to vaccination is weaker than in a healthy population [12]. A possible explanation could be one in which the phenomenon might be a consequence of RBD-independent SARS-CoV-2 recognition. The major aim of the article is to discuss receptor-associated aspects of the SARS-CoV-2 infection mechanism that can contribute to severe COVID-19 development in patients with T2D.

## 2. Expression Profiles of SARS-CoV-2 Receptors

Respiratory droplets and aerosols have been suggested as a primary route of SARS-CoV-2 transmission [13]. Besides the lungs, the presence of SARS-CoV-2 structural components was also observed in the heart, kidneys, liver, and spleen [14]. It has been shown that the SARS-CoV-2 receptor is ACE2, which directly binds to the viral spike (S) protein [15]. Dissociation constant analyses revealed a high binding affinity of ACE2 toward S protein RBD (77 nM). The glycosylation of both ACE2 and the SARS-CoV-2 receptor-binding domain decreased the K_D_ value even to 30 nM [5]. The binding affinity also differed between variants of SARS-CoV-2. The K_D_ values estimated via microscale thermophoresis (MST) measurements were as follows: 27.5 nM, 11.8 nM, 23.1 nM, 21.5 nM, and 5.5 nM for wild-type, alpha, beta, delta, and omicron variants, respectively [16,17]. Several reports have indicated that the involvement of ACE2 in the mechanism of SARS-CoV-2 cell entry is crucial [18,19]. However, the issue is more complex since other factors can influence the functioning of the receptor in vivo. One of them is ACE2 protein cell surface expression.

The transcriptomics approach revealed that the small intestine, colon, duodenum, kidney, testis, gallbladder, and heart are the major sites of ACE2 synthesis in the organism [4]. In the group of organs with consensus expression exceeding 1% of maximum, the pancreas, adipose tissue, oesophagus, and liver should also be mentioned. It has been postulated that the higher expression of *ACE2* in these organs may be responsible for the development of organ-specific symptoms. Some of the literature reports confirm these assumptions. Gastrointestinal symptoms, renal failure, and cardiac injury, to name a few, have been observed in the course of COVID-19 [20,21]. The abundance of ACE2 in the respiratory system is low, limited to ciliated cells of the nasal mucosa, bronchus, and lungs, as revealed by immunochemical analyses [4]. Earlier studies indicated higher synthesis levels [22]. Additionally, negligible amounts of mRNA encoding the enzyme are present in these cells. Since respiratory symptoms are important in the disease course, alternative ways of SARS-CoV-2 cell internalisation have been considered, and several other receptor candidates among cell surface proteins have been suggested. Many candidates have been predicted via in silico analyses; several other methods have also been applied. The issue complexity can be illustrated by the results obtained by Liao et al. in which 293 proteins interacting with SARS-CoV-2 spike protein were identified by mass spectrometry analyses of co-immunoprecipitates in human lung cells only; 42 proteins were membrane-associated, 9 of them could be found at the cell surface [23]. To be considered a receptor, the protein (or other compound) has to meet two basic criteria: it has to reveal the abilities of SARS-CoV-2 binding, and binding has to produce the effective infection. Several particles revealing such abilities, as well as the most essential proteases, are listed in Table 1.

Some of the genes encoding independent receptors for which participation in S binding has been confirmed display high expression in the lungs, especially tyrosine-protein kinase receptor UFO (*Axl*), neuropilin 1 (*NRP1*), transferrin receptor (*TfR*), and glucose regulated protein-78 (GRP78, heat shock protein family A (Hsp 70) member 5—*HSPA5*). Expression data available from the Human Protein Atlas [92]: v23.proteinatlas.org (for appropriate URLs see Supplementary Materials) suggest their responsibility for the observed symptom development characteristic of COVID-19. Application of immunochemistry methods revealed that AXL protein expression is limited only to the lungs, while the presence of TfR has also been detected in the bronchus. NRP1 is expressed in the lungs, bronchus, and nasopharynx epithelial cells.

CD147 is also present in the respiratory system [93]. It is thought to take part in mucosa secretion and remodelling of airways during asthma development [94,95]. It has also been suggested that CD147 may be responsible for the fibrosis progress observed in the course of SARS-CoV-2 infection since the process requires CD147-dependent fibroblast activation [96].

**Figure 1:**
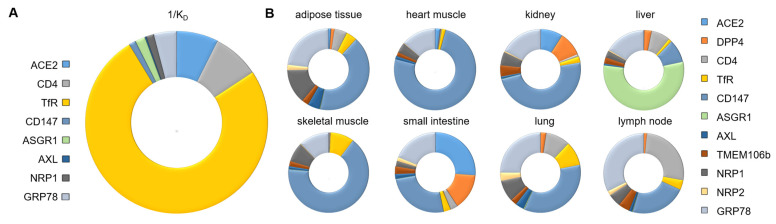
Basic characteristics of selected SARS-CoV-2 receptors. (**A**) Relative affinity of wild-type SARS-CoV-2 spike protein binding (1/K_D_): K_D_ values: ACE2—30 nM [5], CD4—27 nM [56], TfR—2.95 nM [23], CD147—185 nM [36], ASGR1—124.6 nM [61], AXL—882 nM [64], NRP1—166.2 nM [98], GRP78—55.2 nM [40]; in comparison: TMEM106B—20 μM [71]. (**B**) Tissue-specific expression of selected genes encoding SARS-CoV-2 receptors. Expression data available from: Human Protein Atlas [92], v23.0.proteinatlas.org.

All of them are located at the cell surface, bind to the spike protein of SARS-CoV-2 or their binding ability was predicted, and can function as independent receptors promoting viral entry, in some cases as efficiently as ACE2 [64]. Moreover, some of them may promote the favourable cell entry of one of the variants of SARS-CoV-2, as was suggested in the case of NRP1 (omicron) [97]. Since the expression of ACE2 in the olfactory epithelium is low [4], NRP1 is listed as one of the main factors that facilitates SARS-CoV-2 infection, a phenomenon observed in five out of six COVID-19 cases considered in the report [75]. SARS-CoV-2-infected cells also revealed higher expression of NRP1, TMPRSS2 (transmembrane serine protease 2), and furin-encoding genes. The relative tissue expression of genes encoding SARS-CoV-2 receptors and their relative affinity toward SARS-CoV-2 spike protein are presented in Figure 1.

## 3. Receptor-Based Implications for T2D-Associated COVID-19 Severity

One of the effects of obesity is impaired adipocytokine secretion. Lower serum concentrations of adiponectin and increased secretion of pro-inflammatory adipokines, such as TNF-α and IL-6, are observed [99]. It leads to limited insulin sensitivity via insulin receptor substrate 1 (IRS1) Ser/Thr phosphorylation. It results in the withdrawal of glucose transporter 4 (GLUT4) from the cell membrane, initially without affecting the functions of the insulin receptor [100]. However, prolonged action of pro-inflammatory factors results in impaired insulin susceptibility. The phenomenon concerns tissues, where GLUT4 is the predominant glucose transporter and is accompanied by increased secretion of insulin from β-cells [101]. Pro-inflammatory adipokines promote chronic inflammation development, which is associated with activation of NF-κB [102,103]. NF-κB is responsible for the induction of inflammatory mediator expression, including cytokines and chemokines essential in the recruitment of macrophages and certain subsets of T and B cells to adipose tissue. As a result, the number of adipose tissue macrophages (ATMs), usually in the M1 pro-inflammatory polarisation state, increases and may exceed 40% of all cells in adipose tissue [104,105]. Together, β-cells are affected, resulting in reduced insulin secretion [106] and increased hyperglycaemia.

All described disorders influence the functioning of metabolism at the cellular level. It is therefore not surprising that they can affect the functioning of SARS-CoV-2 receptors. Some of the receptors, such as ACE2, CD147, GRP78, integrin α_5_β_1_, and TfR, can be considered high glucose/diabetes inducible, a phenomenon observed in several types of cells [25,39,42,45,50]. Some can be regulated via pro-inflammatory adipokines, such as IL-6 and TNF-α. GRP78, integrin α_v_β_3_, and NRP1/2 (as ACE2 co-receptors) should be included in the group. Besides those mentioned, the functions of CD4 (cluster of differentiation 4), DPP4/CD26 (dipeptidyl peptidase 4), mGluR2 (glutamate metabotropic receptor 2), vimentin, and sialoglycans will be discussed.

### 3.1. ACE2

The ability of a virus to infect a cell should be the result of its receptor abundance at the cell surface and its ability to bind to it, an event necessary to produce effective cell entry. The tissues that show high ACE2/TMPRSS2 expression, since ACE2-dependent SARS-CoV-2 uptake is the most frequently discussed mechanism of infection, should be considered as the prime sites of virus internalisation, whereas tissues and cells where protein synthesis is low should not be susceptible to virus attack. In order to test the hypothesis, analyses that examined the SARS-CoV-2 genome amplification rate in various types of enterocytes with differing *ACE2* expression have been performed. The studies revealed no correlation between these two variables [107], indicating that *ACE2*, displaying the highest expression among human tissues, is not an exclusive SARS-CoV-2 receptor in enterocytes. However, increased expression of *ACE2* may affect the way in which SARS-CoV-2 spreads. The formation of “intercellular extensions” in human lung epithelial cells between infected and non-infected cells, providing the virus with a novel effective route of cell-to-cell transmission, was observed within cells with induced overexpression of *ACE2* [108]. Moreover, the route can also be used to infect cells that are not susceptible to viral attack, since the process may spread infection between various types of cells. It was shown that neurons that were not susceptible to SARS-CoV-2 uptake via endocytosis were directly infected by epithelial cells through nanotubes [109]. The phenomenon may explain COVID-19-associated neurological symptom occurrence and reveal how increased expression of ACE2 may be beneficial for SARS-CoV-2 virulence.

**Figure 2:**
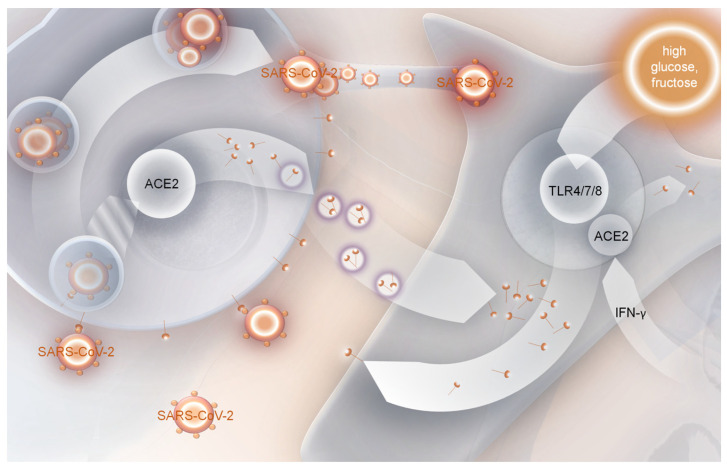
Probable ACE2-dependent mechanism of SARS-CoV-2 infection in tissues with low *ACE2* expression. ACE2—angiotensin-converting enzyme 2, TLR4/7/8—toll-like receptors 4/7/8, IFN-γ—interferon gamma.

Increased expression of ACE2 in diabetes was reported in human heart muscle cells and in the kidneys [25,110], suggesting induced susceptibility to SARS-CoV-2 infection. On the other hand, there was no difference in ACE2-encoding mRNA expression in lung tissue between diabetic (T2D) and non-diabetic patients, but protein amounts in the alveolar and bronchial epithelium in diabetes were increased [111]. It is not fully understood what causes increased content of the protein in cells, but some clues have been provided by studies of monocytes and macrophages, both blood and lung associated. The expression of *ACE2* in monocytes is very low; several studies reported the lack of gene expression in cells of the immune system, and even though some cells were resistant to pseudovirus attack, alveolar macrophages were still susceptible to SARS-CoV-2 infection [112,113]. A study by Yao et al. indicated that monocytes are infected by the virus only when ACE2 and TMPRSS2 are present at the cell surface [114]. Although synthesis of both genes was not observed, ACE2 was still present in the cytoplasm of the cells. It was postulated that ACE2 could be provided by exosomes, which can bear this protein within their protein load. For the migration of ACE2 into the cell membrane, activation of TLR4/7/8 (toll-like receptor family 4/7/8) upon viral proteins or single-strand RNA presence is required [114]. The induction of TLR4-encoding mRNA synthesis along with increasing homeostatic model assessment (HOMA-IR) score was reported earlier in peripheral blood leukocytes [115]. A similar phenomenon was observed in retinal vascular endothelial cells due to high glucose, where TLR4 was also activated [116]. Moreover, increased expression of TLR7 was reported in mice retinal pigment epithelial cells treated with fructose [117], as well as TLR8 synthesis in subcutaneous adipose tissue in response to obesity [118]. It was correlated with increased pro-inflammatory cytokine synthesis. Together, these results show that even cells with minimal expression of the receptor could be the target of SARS-CoV-2. The phenomenon could also be a reason for the diabetes-related increase in ACE2 membrane concentration in lung tissue (Figure 2).

The functioning of toll-like receptors may also be more complex. TLRs are involved in IFN type I signalling regulation. It was shown that both decreased or increased abundance of IFN active forms can be associated with severe COVID-19 and increased mortality. Many reports have indicated the presence of high serum levels of type I IFN as well as IFN-γ in critically ill patients [119,120]. Moreover, IFN-α/β serum concentration was positively correlated with TNF-α but negatively with CD3^+^, CD4^+^, and CD8^+^ T cells. Dendritic cells and macrophages were shown to be responsible for cytokine secretion. Simultaneously, genetic disorders such as X-linked TLR7 deficiency, causing decreased reaction to stimulating factors, as well as mutations in genes of the type I IFN pathway, causing decreased type I IFN levels or IFN I susceptibility, can also affect COVID-19 outcomes [121,122]. It reveals that strict control of cytokine secretion and activation is required to maintain proper functioning of the immune system after SARS-CoV-2 infection. Diabetes can influence the homeostasis. It was shown that levels of IFN-γ may be increased during T2D development [123]. An interesting aspect of the issue is diverse IFN expression profiles in subsequent sections of the respiratory track in mild or severe COVID-19. Some of the expressed IFN species (λ1/3) revealed protective roles against SARS-CoV-2 via induction of IFN-stimulated genes in mild COVID-19. On the other hand, overexpression of IFN-α/β or IFN-λ2, accompanied by a reduction of the IFN-λ1/3 synthesis rate, especially in the lungs, induced apoptosis and was associated with severe disease development. Additionally, the correlation between expression levels of the mentioned proteins and their serum concentrations was not obvious [124].

Sodium-dependent imino transporter 1 (SIT1), belonging to the SLC6 transporter family (SLC6A20), is another factor that may influence ACE2 abundance at the cell surface. The protein functionally interacts with ACE2. It was shown that in HEK293 cells overexpressing the protein, the majority of ACE2 was localised within the cytosol. The phenomenon decreased simultaneously the ability of cells to bind to the RBD of SARS-CoV-2 omicron variants [125]. It was demonstrated earlier that *SLC6A20* can also be involved in T2D development. Decreased mRNA expression was observed in the kidney proximal tubules of T2D monkeys, which caused disorders of some metabolite concentrations (glycine, proline, or betaine) in urine. The same observations were considered diabetic mice [126]. *SLC6A20* is expressed in type 2 human alveolar pneumocytes; expression in other cells is low [127]. The *SLC6A20* gene is located in the locus 3p21.31, which contains 5 other genes: *CXCR6* (CX motif chemokine receptor type 6), *CCR9* (CC motif chemokine receptor 9), *XCR1* (XC motif chemokine receptor 1), *LZTFL1* (leucine zipper transcription factor-like 1), as well as *FYCO1* (FYVE and coiled-coil domain autophagy adaptor 1) [128]. Twenty variants of this cluster have been identified [129]. Genome-wide association studies (GWAS) on single nucleotide polymorphism (SNP) revealed a strong correlation between the *SLC6A20* gene regulatory region (allele GA of rs11385942) and the respiratory failure observed in severe COVID-19, although the allele can be characterised by decreased expression of *CXCR6* but increased synthesis rate of *SLC6A20* and *LZTFL1* [128]. It could suggest a lower abundance of membrane-bound ACE2 (in the context of the proposed SIT1 mechanism of action) and a lower susceptibility of respiratory track cells to SARS-CoV-2 infection, unless other unrecognised mechanisms of SARS-CoV-2 cell uptake upregulated by the gene’s increased expression dominate over the ACE-2-dependent way of infection in alveolar cells or some other factors are engaged.

In 2024, Loktionov et al. studied which of the SNPs involved in severe COVID-19 development may also be associated with increased risk of obesity/T2D. Strong correlations with obesity were revealed for rs17713054 *SLC6A20–LZTFL1* and rs7949972 *ELF5*, whereas the probability of participation of rs9636867 *IFNAR2* in T2D development was high. The functioning of these SNPs was explained by their participation in the influx of cells of the immune system into adipose tissue (chemokine receptors), reduced *CAT* (catalase) expression, and simultaneously increased leptin serum levels (*ELF5*—E74 like ETS transcription factor 5), or mediated by interferon α/β receptor 2 (*IFNAR2*) onset of β-cell dysfunction, as well as insulin resistance development via the JAK-STAT pathway [130]. Moreover, it was observed that, besides obesity, the *SLC6A20* variant (allele A of rs13062383) can also be associated with T2D development in humans [131].

There are some other genes that can be implicated in the worsening of outcomes in COVID-19. One of them is *TYK2* (tyrosine kinase 2) with decreased expression due to polymorphism of its promoter region, which is correlated with the progress of T1D as well as T2D, with odds ratios exceeding 2 [132]. Viruses may be implicated in T2D development via TYK2, which is involved in the regulation of cytokine signalling, with IL-6 among others. It seems to be true since COVID-19 patients also reveal decreased expression of the gene [133]. On the other hand, these phenomena may also be a reason for poor outcomes in COVID-19 among patients with diabetes.

Polymorphisms of *ACE2* should also be discussed. Studies have revealed the diversified influence of *ACE2* variants on the COVID-19 course. Certain variants of the gene (allele AA of rs2285666, TT of rs2074192, and GG of rs4646174, or TT alleles of both rs4646156, and rs2158083) may contribute to more severe COVID-19 with OR values ranging from 2.12 to 1.84 [134]. Some other reports have indicated protective roles for some identified SNPs [135,136]. However, it was also concluded that *ACE2* polymorphism and diabetes were independent risk factors [134].

On the other hand, several other explanations have been proposed to justify how cells with very low or no *ACE2* expression are infected. Han et al. suggested GRP78 as a receptor for SARS-CoV-2 cell entry [40]. Junqueira et al. revealed that only several per cent of blood and lung-resident monocytes and about 8% of lung macrophages were infected by the virus in patients with COVID-19. Upon infection, activation of caspase 1 occurred via the NLR family pyrin domain containing 3 (NLRP3, recognising membrane damage) and absent in melanoma 2 (AIM2, recognising cytoplasmic DNA) inflammasome activation, which led to monocyte pyroptosis. Viral replication did not occur. SARS-CoV-2 cell uptake was antibody dependent, mediated by IgG Fc receptors (FcγR), another type of SARS-CoV-2 receptor, since antibodies against two FcγRs, CD16 and CD64, blocked the ability of the virus to infect cells. However, another mode of inflammasome activation should also be taken into account, since the lung macrophages with activated inflammasomes were four times more numerous than infected [68]. As the authors emphasised, some part of macrophage infection could be ACE2-related if ACE2 is present in lung macrophages, which was not observed by them but was discussed earlier [114]. These data also indicate the connection between a monocyte/macrophage SARS-CoV-2 infection mechanism and diabetes. Although CD64 and CD16 are not diabetes inducible, activation of the NLRP3 inflammasome was observed in both T1D and T2D, where it is considered a major factor engaged in the chronic inflammation present in hyperglycaemia [137]. The participation of other receptors has also been discussed in the case of infection of respiratory system tissues.

There are many other factors affecting the functioning of ACE2 upon SARS-CoV-2 infection. The adipose tissue might be a good example. It was observed that *ACE2* expression in adipocytes is higher than in lung tissue [138]. However, even within adipose tissue, *ACE2* expression is diversified; higher levels have been found to be related to the development of insulin resistance in epicardial and visceral fat cells rather than in subcutaneous fat cells [138,139]. However, the gene synthesis rate decreased in diabetic patients [26]. It could suggest a lower susceptibility to SARS-CoV-2 infection. But the development of diabetes is usually associated with obesity, which increases the number of fat cells and can mimic the effect of decreased *ACE2* expression. Moreover, diabetes can be characterised by a state of chronic inflammation associated with an influx of immunological cells, from which each type has its own unique susceptibility and/or mechanism of response to SARS-CoV-2 infection, modulating the functioning of the entire tissue. It was reported that *ACE2* expression in pneumocytes may be induced by type 2 interferon alpha and interferon gamma (IFN-α2 and IFN-γ) secreted by lung-associated macrophages [140]. Although it was not studied, the phenomenon could possibly affect the functioning of adipose tissue. ACE2 is also an active enzyme that takes part in the synthesis of anti-inflammatory peptides, such as angiotensin 1–7 [141]. The compound may reduce visceral adipocyte mass and size as well as ER stress markers concentrations, mimicking the characteristic disorders usually observed in prediabetic patients [142]. It restores the adipocytokine balance, reducing elevated TNF-α levels, which is directly involved in insulin resistance development [143]. All together, it shows the complexity of the discussed issue. Some other aspects of ACE2 functioning will also be discussed in subsequent chapters.

### 3.2. CD147

CD147/basigin, originally found in the membrane of tumour cells, may induce some metalloproteinase activities. It is also considered to be a factor that promotes cardiac fibrosis [144], and a plasma level of the protein is correlated with obesity, hyperglycaemia, and advanced glycation end products (AGEs) [39]. It is inducible by AGEs and high glucose concentrations in monocytes [145] and may be implicated in renal function [146]. CD147 may also regulate pro-inflammatory pathways since decreased expression of the protein induces apoptosis in synovial fibroblasts. It also inhibits NF-κB-dependent proinflammatory cytokine secretion [147], which indicates the participation of CD147 in the development of the inflammation typical in rheumatoid arthritis.

The protein is involved in characteristic COVID-19 platelet activation since monoclonal antibodies against CD147 inhibited the process [148]. Wang et al. reported that Meplazumab, another CD147-blocking antibody, is an efficient SARS-CoV-2 amplification reducer [36]. Moreover, the analyses showed that CD147 binds to SARS-CoV-2 RBD with K_D_ = 185 nM. Internalisation of the virus was supposed to occur via the endocytosis pathway. However, several reports have called the proposed receptor function of the protein into doubt. There was no correlation in expression between the CD147 gene and viral genome abundance in various enterocyte species, along with the ACE2 and DPP4 genes [107], although the presence of such an association was reported in lung epithelial adenocarcinoma cells [37]. A study by Shiltz et al. failed to find evidence of direct CD147–spike protein interactions [149]. Moreover, New Zealand White rabbit-derived polyclonal anti-basigin IgG, other than Meplazumab, had no influence on SARS-CoV-2 infection effectiveness [150]. Even if CD147 may not be the SARS-CoV-2 receptor sensu stricto, alternative paths engaging this protein may exist in the viral cell entry process. It was shown that an extracellular ligand for CD147, cyclophilin A (CyPA), seemed to be implicated in SARS-CoV-2 infection since its inhibitors reduced the viral genome copy number via protecting against CyPA binding to the C-terminal domain of the viral nucleocapsid [38]. The protein can also modulate ACE2 levels, and the expression of both is downregulated upon SARS-CoV-2 infection in lung epithelial cells [37], which suggests an indirect function of the protein in the modification of SARS-CoV-2 virulence. The elucidation of which of the proposed mechanisms is the most accurate needs further studies.

### 3.3. GRP78

The level of GRP78 is associated with obesity and T2D in human blood [151]. Moreover, a high GRP78 membrane surface concentration in high glucose-treated vascular endothelial cells was also observed [42]. The basic function of the protein is dual. It is responsible for the control of protein folding in the endoplasmic reticulum as well as senses stress signals [152], acting as a transcriptional regulator under stress conditions [153]. The interaction of GRP78 with integrin β_1_ at the cell surface may activate the synthesis of proteins of the extracellular matrix under high glucose stimulation in the pathogenesis of diabetic nephropathy [41]. The interaction activates focal adhesion kinase, which affects profibrotic PI3K/AKT (phosphoinositide 3-kinase/AKT Ser/Thr kinase) signalling pathway functioning. Besides high glucose, TNF-α and IL-1β are inducers of GRP78 expression. The phenomenon was observed in rheumatoid arthritis synoviocytes and, similarly to CD147, reduced expression of the GRP78 gene increased the synoviocyte apoptosis rate [154]. However, another mechanism of GRP78 functioning in cancer cells exists. The presence of M2 macrophages in the tumour microenvironment results in elevated GRP78 expression in cancer cells. Direct interactions of the protein with Janus kinase 2 (JAK2) and signal transducer and activator of transcription 3 (STAT-3) cause the phosphorylation of the latter and induce TNF-α and IL-1β secretion, which in turn facilitate tumour metastasis [155]. TNF-α, IL-1β, and IFN-γ are also responsible for membrane incorporation of the protein in β-cells. Translocation occurs upon the induction of ER stress following cytokine stimulation. Membrane-bound GRP78 was determined in 94% of early-apoptotic cell species of murine MIN6 cells, appearing after exposure. The process depended on transport via the Golgi apparatus. Surface GRP78 functions were associated with expression of several pro-apoptotic markers [156]. A similar phenomenon was observed in human retinal microvascular endothelial cells [157]. The process was accompanied by increased permeability of the cell layer.

The presented results indicate increased membrane-bound fraction abundance of GRP78 under inflammation. Since it is also considered as one of the proteins essential for SARS-CoV-2 binding at the cell surface, the existing mechanism may affect the effectiveness of virus cell entry. Elevated expression of the protein under SARS-CoV-2 infection [158] may be one of the premises indicating the participation of the protein in the SARS-CoV-2 infection process. The latest studies revealed that application of YUM70, a hydroxyquinoline analogue and inhibitor of GRP78, interrupts SARS-CoV-2 cell entry into Vero E6 cells (African green monkey kidney cell line). Moreover, the pathological changes observed as a result of virus infection in lung tissue were reduced in mice treated with the compound [159]. The same effect was obtained after knockdown of protein expression [158].

The possibility of forming a triple complex of GRP78 with viral spike protein and ACE2 via the substrate-binding region was reported [160]. Simultaneously, reduced expression of GRP78 was associated with decreased concentration of ACE2 at the cell surface. The same phenomenon was observed after blockade of the cell surface-associated GRP78 by antibodies, which together may suggest that the protein may function as an ACE2 co-receptor or, by having an impact on *ACE2* expression, may regulate the SARS-CoV-2 cell internalisation rate. On the other hand, cell surface GRP78 concentration in monocytes and macrophages is associated with the inflammation observed in the COVID-19 course. THP-1 cells with high GRP78 and low ACE2 expression were able to uptake pseudovirus-containing spike protein. Moreover, it was shown that GRP78 interacts directly with the spike protein of wild-type SARS-CoV-2 with a dissociation constant K_D_ = 55.2 nM. The affinity toward omicron variant spike protein was higher (K_D_ = 27.2 nM) [40]. Another study revealed that GRP78 may also independently interact with other viruses, such as Newcastle disease virus, producing an effective infection [161].

### 3.4. DPP4/CD26

One of the proteins that was originally proposed as the receptor for SARS-CoV-2 is dipeptidyl peptidase 4/cluster of differentiation 26 (DPP4/CD26). This suggestion was based on the protein binding ability toward MERS-CoV spike protein [162], computational docking analyses with crystal structures of CD26 and SARS-CoV-2 spike protein, and the presence of the protein on the cell surface [163]. Although well established in computational predictions, the protein’s participation in SARS-CoV-2 spike protein binding is still elusive [15,43,164]. However, there are several indirect observations that prompt a more detailed discussion. Circulating DPP4/CD26 has been proposed as a novel adipokine and a marker of visceral obesity and insulin resistance [44], revealing a strong association with pre-diabetic disorders. DPP4 in soluble form appears in circulation due to shedding, the process associated with the cleavage of extracellular domains of proteins bound to the cell membrane by specific proteases. The phenomenon was observed in ovarian cancer cells, and matrix metalloproteinases 10 and 13 (MMPs 10 and 13) were responsible for the cleavage. Simultaneously, a strong induction of *DPP4* expression was observed under hypoxic conditions [165]. A relatively high level of hypoxia is observed in adipose tissue in obesity, which is one of the factors that lie at the core of T2D development. Chowdhury et al. studied the phenomenon. They observed decreased expression of cell surface-associated DPP4 in pre-adipocytes under hypoxic conditions. Insulin stimulation of the cells caused a rapid increase in protein expression under both hypoxic and normoxic conditions. The increase in hypoxia was almost two times higher than that in cells cultured in medium with normal oxygen content. The phenomenon was accompanied by an increased concentration of the protein in soluble form due to the shedding process. However, it was observed that high membrane expression of DPP4 occurs only in pre-adipocytes, which decreases along with differentiation progress [166]. It seems important in the context of the discussion of diabetes-associated COVID-19 severity since hypoxia inhibits adipogenesis [167].

DPP4/CD26 inhibitors (gliptins) have found application in the treatment of hyperglycaemia since they inhibit the degradation of glucagon-like peptide-1 (GLP-1) and glucose-dependent insulinotropic polypeptide (GIP) hormones [168]. They also revealed antifibrotic and anti-inflammatory properties [169,170]. DPP4/CD26 is a serine endopeptidase ubiquitously expressed in different cell types, in adipose tissue, as well as in immune cells (macrophages, B and T cells, or NK), among others [171,172,173]. An important property of the enzyme in COVID-19 progression is its ability to activate the processes of the immune response. It was shown that circulating DPP4/CD26 activated ERK1/2 and NF-κB in vascular smooth muscle cells. It also induced cell proliferation, and increased IL-6 and interleukin 8 (IL-8) secretion accompanied the process [174]. DPP4/CD26 may be involved in the N-terminal processing of precursors of several cytokines and chemokines; the cell surface DPP4/CD26 fraction may also induce T cell proliferation [175,176]. Moreover, CD26 deficiency triggers M2 macrophage differentiation [172], and DPP4/CD26 inhibitors can decrease the risk of autoimmune disease occurrence in the course of T2D, as was shown in clinical studies [177]. Together, these findings suggest the participation of the protein in the development of a hyperimmune response characteristic to COVID-19, called the “cytokine storm” observed in T2D patients, one of the prime causes of COVID-19 mortality. In fact, there are several studies indicating the positive impact of application of DPP4 inhibitors in therapy on the clinical outcomes of both diabetic and non-diabetic patients with COVID-19. Application of sitagliptin reduced C-reactive protein (CRP), IL-6, and ferritin levels in comparison to standard methods in clinical trials, but the small cohort size (40 participants), might have influenced the obtained results [178]. It should be mentioned that reports exist indicating no correlation of COVID-19 mortality with DPP4 inhibitor treatment [179]. However, a meta-analysis provided by Zein and Rafaello based on 11 studies revealed decreased mortality in the group of COVID-19 patients who were treated with DPP4 inhibitors [180].

### 3.5. TfR

Transferrin receptor (TfR) is a protein expressed in the liver, lungs, endometrium, appendix, and bone marrow (expression data available from the Human Protein Atlas [92]: v23.proteinatlas.org; for appropriate URL, see Supplementary Materials). The expression is high in the liver, but low or even negligible in other tissues. The principal function of the protein is the cellular uptake of transferrin-bound iron. It is a homodimer built up of subunits connected by disulfide bridges. Both subunits consist of a large C-terminal extracellular globular domain and two smaller transmembrane and intracellular domains [181]. The extracellular domain of each subunit binds one transferrin molecule, and transferrin is taken up via endocytosis [182]. In 2022, Sokolov et al. reported that SARS-CoV-2 replication in Vero cells can be inhibited by ferristatin II [183]. It put the transferrin receptor in the circle of putative SARS-CoV-2 receptors. Further investigations by Liao et al. revealed that TfR binds to the spike protein with the highest affinity (K_D_ = 2.95 nM) among all known SARS-CoV-2 receptors. Virus internalisation occurs via endocytosis, and the process is inhibited by transferrin, soluble TfR, some peptides (SL8 and QK8), and anti-TfR antibodies blocking the contact site of membrane-bound TfR. Mice expressing human TfR, which originally had not been infected, were susceptible to SARS-CoV-2 infection [23].

**Figure 3:**
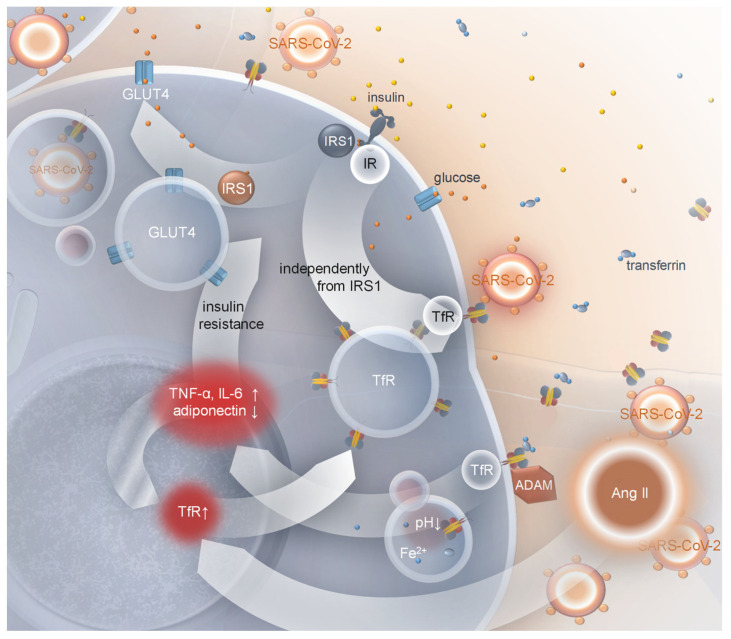
Regulation of membrane translocation of transferrin receptor (TfR) in adipocytes. IR—insulin receptor; IRS1—substrate of insulin receptor 1; GLUT4—glucose transporter 4; ADAM—a disintegrin and metalloproteinase domain-containing protein; Ang II—angiotensin II; arrows correspond to changes of synthesis/secretion rates of specified proteins or changes of pH; yellow dots: insulin molecules; orange dots: glucose molecules; blue dots: ionised iron.

One of the aspects of the pathogenesis of type 2 diabetes mellitus is iron imbalance, the manifestation of which may be ferroptosis, iron-regulated cell death. It was shown that ferroptosis may be involved in diabetic β-cell damage [184]. The development of insulin resistance may also be associated with iron metabolism disorders since diabetic patients display high serum transferrin concentrations [185]. It may also influence serum adiponectin concentration by affecting the functioning of adipocytes. Mice fed a high-iron diet had a higher serum concentration of insulin resistance-associated adipokines [186]. It was observed that the expression of TfR is upregulated in the kidneys of diabetic rats [47]. Important observations were provided by Davis et al. [46]. The authors showed that TfR is translocated from the cytoplasm into the cell membrane under insulin signalling in rat adipocytes. The same type of membrane-bound TfR abundance regulation was discovered in human adipocytes [187]. Co-studies of insulin-induced membrane incorporation of TfR and GLUT4 revealed that the phenomenon is regulated in a different manner. The rate of TfR incorporation was three times higher, and only several proteins regulating the process were common for both; others were exclusively connected with GLUT4 translocation [188]. It is not known whether insulin resistance influences the TfR membrane incorporation rate. However, it is well established that insulin resistance affects the number of membrane-incorporated insulin receptors, and since insulin is considered a major regulator of TfR translocation, the membrane-bound fraction of the protein should not increase, even if hyperinsulinemia accompanies insulin resistance, unless some other factors are engaged. The reaction of various tissues to exogenous TNF-α was observed, a cytokine which, along with chronic inflammation, blocks GLUT-4 membrane translocation. TNF-α increased expression of TfR in the vascular endothelium, as well as in ventral mesencephalic neurons (along with IL-1β) and fibroblasts [189,190,191], but not in A549 cells [192]. Moreover, TfR gene expression was angiotensin II (Ang II) inducible, suggesting that Ang II may influence iron imbalance in the pathogenesis of metabolic syndrome [193]. It was also induced by IFN-γ, at least in monocytes [194], which could be associated with the chronic inflammation development observed in diabetes. Simultaneously, TNF-α and IL-1 showed inhibitory effects. Together, it suggests the existence of other means, besides iron load and insulin, by which various cells can control the membrane-bound fraction abundance of the protein (Figure 3).

Another aspect of receptor functioning is its soluble form. According to meta-analyses, diabetes is accompanied only by a slight increase in TfR serum level [195]. The inhibitory effect of soluble TfR on SARS-CoV-2 virulence should therefore be limited. Anyway, further studies are needed to provide an elucidation of the character of the possible participation of TfR in diabetes-induced severe COVID-19.

### 3.6. Integrins α_5_β_1_ and α_v_β_3_

The function of integrins in SARS-CoV-2 binding was originally predicted based on the observation of the presence of the RGD motif (403–405: Arg-Gly-Asp) in close proximity to the ACE2-binding region on the spike protein surface, which is a distinguishing feature for SARS-CoV-2 [196]. Of the 24 known heterodimeric integrin receptors, seven possess the ability to bind to RGD. Further studies have revealed that two of them, α_5_β_1_ and α_v_β_3_, may act as SARS-CoV-2 receptors in vitro [48,52]. The basic function of integrin receptors is to participate in interactions with the extracellular matrix as well as with other cells, mediating bi-directional trans-membrane signalling [197]. However, individual integrin receptors possess more specific functions. Integrins are ubiquitously expressed in human tissues. It has been frequently observed that high glucose may induce the expression of some of the integrins, with α_5_β_1_ among others. High glucose increased the strength of the association between vascular endothelial cells and extracellular matrix elements. Along with α_5_ and β_1_ subunits, expression of fibronectin, an RGD motif-containing α_5_β_1_ substrate, was also induced [50]. Moreover, a higher abundance of the β_1_ subunit was observed in the microvessels in mesangial and glomerular endothelial cells of diabetic patients. It produced firmer cell adhesion, which was associated with increased collagen IV deposition in the extracellular space [51]. The effect of high glucose was protein kinase C (PKC) dependent. Together, it indicates that hyperglycaemia may affect the interaction strength of several cell types with their basement membranes. It was also shown that selective deletion of the β_1_ subunit in murine adipocytes may cause systemic insulin resistance [198]. Activation of the α_5_β_1_ integrin receptor upon SARS-CoV-2 infection causes several events in endothelial cells. Activation of NF-κB, along with increased expression of pro-inflammatory cytokines (TNF-α, IL-1β, and IL-6), ACE2, or coagulation factors was observed [48]. These effects were accompanied by increased monolayer permeability and could be suppressed by α_5_β_1_ inhibitors.

Less is known about α_v_β_3_ functioning in diabetes. It was revealed that the protein, upon osteopontin binding, may promote aerobic glycolysis via focal adhesion kinase activation in glioblastoma cells [199]. The integrin receptor may also be responsible for diabetic nephropathy development in pigs [200]. Downregulation of the integrin receptor was observed in murine endothelial progenitor cells upon an atherogenic diet [201]. On the other hand, immunohistochemical staining revealed higher expression of α_v_β_3_ within the retinal tissues of diabetic guinea pigs [202]. Moreover, integrin was TNF-α inducible in chondrosarcoma and in gastric cancer cells (α_v_) [203,204]. The protein was also involved in TNF-α-induced endothelial cell migration [205], since higher expression of the protein was observed within migrating cells. Integrin α_v_β_3_ functions in endothelial cells as a SARS-CoV-2 receptor, independently of ACE2, promoting virus cell entry via endocytosis. The antibody against the protein inhibited the process, but the vaccine-derived anti-spike antibodies did not protect against the integrin α_v_β_3_ and the RGD motif of spike protein interactions [53].

### 3.7. CD4

Pontelli et al. reported an effective SARS-CoV-2 infection of human monocytes and B and T lymphocytes [206]. An increased apoptosis rate was observed among infected cells. Since CD4 is a co-receptor of the HIV virus [207], Shen et al. decided to verify if CD4^+^ T cells can be infected by SARS-CoV-2 in vitro. TMPRSS2 inhibitors, ACE2 knockdown, or blocking antibodies were applied. The results showed that the cells were susceptible to virus infection, and the infection mechanism was independent of the ACE2/TMPRSS2 system. Due to infection, extensive apoptosis of T cells occurred [208]. Similar effects were observed by Brunetti et al. In order to identify the potential receptor, they isolated CD4^+^ and CD8^+^ positive T lymphocytes from peripheral blood and exposed them to SARS-CoV-2. It was shown that only CD4^+^ T cells were infected by the virus. Moreover, direct interactions between the S protein as well as the RBD of SARS-CoV-2 and CD4 were observed, with K_D_ values equal to 27 and 22 nM, respectively. CD4 was not an exclusive receptor in helper CD4^+^ lymphocytes since slight expression of ACE2 and TMPRSS2 could be detected in the cells. Although inhibitors of both ACE2 and TMPRSS2 reduced the viral load, it was proposed that CD4 may act as an independent receptor since direct interactions between CD4 and ACE2 were not recognised [56]. The infection led to cell dysfunction and death, accompanied by the release of IL-10 via cAMP-responding element binding protein 1 (CREB-1) phosphorylation. It was suggested earlier that serum levels of IL-10 along with interleukin 1Ra (IL-1Ra, synthesized in monocytes and keratinocytes) and C-C motif chemokine ligand 5 (CCL-5) at early stages of progression may predict the severity of COVID-19 [209,210]. On the other hand, apoptosis of infected cells may be responsible for the lymphocytopenia state characteristic of the COVID-19 course. How can the receptor contribute to severe COVID-19 induction in diabetic patients? Miya et al. provided relevant insight into the topic. An almost 14% increase in the number of CD4^+^ T cells in the group of T2D patients was observed. An increased rate of CD4^+^/CD8^+^ T cells was also reported in both diabetic and non-diabetic patients 120 min after glucose intake during the oral glucose tolerance test (OGTT) [211], suggesting that glucose can influence the proportion of circulating T cell subsets. Simultaneously, it was observed that hyperglycaemia significantly increases the ratio of helper CD4^+^ T cells to all CD4^+^ T cells (from 4.5 to 8.5%) in peripheral blood [57]. A similar induction could also be observed in patients with abdominal obesity. The described results suggest that hyperglycaemia may influence the number of SARS-CoV-2-infected cells in the immune system, which may contribute to the development of the cytokine storm characteristic of severe COVID-19.

### 3.8. mGluR2

The development of diabetic depression may be a possible connection between mGluR2 functioning and hyperglycaemia. Induction of the disorder by high glucose or corticosterone increased mGluR2/3 expression and resulted in damage to a rat hippocampal neurovascular unit model [212]. A study focused on the influence of maternal diabetes on the functions of the rat offspring nervous system revealed increased expression of the receptor in the lateral geniculate body [60] and in the inferior colliculi of neonatal rats [213]. One of the effects of stimulation of mGluR2 in rat microglial cells is TNF-α- and TNF receptor 1-dependent activation of caspase 3 in cerebellar granule neurons in vitro [214].

The expression of mGluR2 is not exclusively associated with the central nervous system. Low mRNA expression has been observed in human testis, skin, skeletal muscle, and adipose tissue (expression data available from the Human Protein Atlas [92]: v23.proteinatlas.org; for URL, see Supplementary Materials). The presence of the protein is also observed in the hearts of rats [215]. Lack of data concerning the impact of hyperglycaemia on receptor expression or functioning in human tissues limits the possibility of comprehensive discussion of mGluR2 engagement in diabetes-associated severe COVID-19 progression. Moreover, the studies have been usually focused on mGluR2/3, which separately showed distinctive effects on the studied cells.

### 3.9. ACE2 Co-Receptors

#### 3.9.1. NRP1/NRP2

A protein co-expressed with ACE2 and TMPRSS2 that induces SARS-CoV-2 virulence is NRP1. The protein does not reveal receptor activity in the absence of ACE2, but application of specific inhibitors showed reduced ability of SARS-CoV-2 to infect ACE2-expressing cells. Moreover, the process depended on furin cleavage of the spike protein [75]. Circulating NRP1 is elevated in T2D-associated hypoglycaemia, but it is not known if the phenomenon affects the risk of SARS-CoV-2 infection [76]. However, NRP1 cell surface expression may be associated with diabetes and metabolic syndrome as well as obesity through oxidative stress-related processes. Studies by Gaddis et al. revealed increased NRP1 expression in mice CD4^+^ T cells upon oxLDL exposure. NRP1-expressing cells displayed a higher ability to secrete IFN-γ, which was associated with atherosclerosis development [216].

Since NRP1 is highly expressed in the olfactory epithelium, much attention has been put onto studies of protein functions in the SARS-CoV-2 infection process. It was shown lately that the gene encoding neuropilin 2 (NRP2), an isoform of NRP1, is highly inducible via co-stimulation with TNF-α and IL-1β in the MH7A human rheumatoid fibroblast-like synoviocyte line, but also in the MRC5 human foetal lung fibroblast-like cell line, however to a lesser degree. The effect can be suppressed by anti-TNF-α antibody. Simultaneously, *NRP1* expression increased only slightly [78]. The effect was originally assigned to COVID-19-associated inflammation but might also be related to chronic inflammation, one of the factors promoting diabetes development. Moreover, expression analyses revealed that suppression of *NRP2* reduced the SARS-CoV-2 genome proliferation rate in cytokine-exposed cells, suggesting that NRP2 may also function as a receptor or auxiliary receptor of SARS-CoV-2, but further details are not recognised.

#### 3.9.2. Vimentin

Vimentin (VIM) was identified as a potential SARS-CoV-2 receptor via mass spectrometry analysis of proteins with S protein RBD-binding ability in human umbilical vein endothelial cell lysates [79]. Simultaneously, it was shown that the protein is able to bind to ACE2, and the function of the ACE2 co-receptor in facilitating SARS-CoV-2 infection was proposed. VIM is a fibrillar protein that is built up of filaments consisting of tetramers as a basic structural feature. The protein determines cell shape and participates in organelle anchorage. It may also be associated with the cell membrane as well as secreted into the extracellular space, as reviewed in 2023 by Arrindell and Desnues [217]. The protein is able to bind to SARS-CoV-2, although virus internalisation is ACE2 dependent since the pseudo-virus infection rate of HEK-293 cells (from the kidneys of a human embryo) expressing VIM alone was only slightly increased compared to control cells [79]. The co-expression of ACE2 with VIM produced the most effective virus internalisation. A similar effect was observed in A549 cells expressing ACE2 treated with purified vimentin, indicating that the extracellular fraction may also be involved in the process. ACE2 and VIM did not compete with each other in RBD binding. VIM is ubiquitously expressed in human cells, with the highest representation in ovary and adipose tissue (expression data available from the Human Protein Atlas [92]: v23.proteinatlas.org; for URL, see Supplementary Materials). An increase in protein secretion has been observed in adipocytes upon oxLDL stimulation, as well as in vascular endothelial cells cultured in high glucose-containing medium [80,81].

#### 3.9.3. Sialic Acid

Sialoglycans on the cell surface function as SARS-CoV-2 receptors, with K_D_ values (monomeric forms) ranging from 100 to 200 nM. They compete for the binding site of the SARS-CoV-2 RBD with heparan sulphate. A 3-time decrease in SARS-CoV-2 infectivity in ganglioside-depleted HEK-ACE2^+^ cells was observed. Simultaneously, ACE2 was necessary for infection to occur [82]. It suggests that sialylated gangliosides can function as ACE2 co-receptors, and both components are needed for effective SARS-CoV-2 cell entry. It was observed that cell surface monosialylated gangliosides are senescence inducible in arterial endothelial cells [84]. Higher blood sialic acid levels were also observed in diabetic patients [83].

### 3.10. Other Receptors and Co-Receptors of ACE2

There are several other proteins that may function as receptors of SARS-CoV-2. The participation of CD209L/L-SIGN and CD209/DC-SIGN (lung cells or macrophage-related lectins, respectively) [67] and transmembrane protein 106B (TMEM106B) [71] in S protein binding has been shown, but the lack of data concerning tissue expression changes in diabetes affects the ability to discuss their possible functions in the development of diabetes-induced severe COVID-19. Some of the proposed receptors may act as SARS-CoV-2 virulence inducers responsible for the severe COVID-19 cases related to diabetes mellitus, at least in cells which proved to have a higher synthesis rate of these proteins (discussed earlier). On the other hand, asialoglycoprotein receptor 1 (ASGR1), Niemann–Pick disease, type C1 (NPC1), and AXL, as well as co-receptors of ACE2, such as heparan sulphate and scavenger receptor, class B type 1 (SR-B1), are downregulated in diabetes [63,66,73,86,91]. It suggests that diabetes may also induce a protective effect against COVID-19 development. Such an effect was observed in vascular endothelial cells cultured under hypoxic conditions in vitro. Expression of both *Axl* and *HIF-1α* (hypoxia-inducible factor 1-alpha chain) was inhibited by high glucose [218]. Simultaneously, studies of adipose tissue revealed that the deletion of *Axl* may protect against obesity caused by diet [219]. Inhibition of AXL induced thermogenesis in the white and brown adipose tissues of mice. Although its direct role in adipogenesis was not proved, it was shown that the expression profile of growth arrest-specific 6 (Gas6) protein—an AXL ligand—resembled that of adiponectin, revealing a constant decrease caused by developing adipose tissue inflammation [220]. These observations suggest the role of AXL in homeostasis maintenance in adipocytes, also in response to high glucose. Data concerning the functioning of the receptor in other tissues in high/low glucose conditions reveal various effects. Although glucose may modify AXL-ligand interactions in vascular smooth muscle cells and contribute to the induction of some of the signalling pathways in a ligand-dependent manner, it does not affect the protein membrane concentrations that could up- or downregulate SARS-CoV-2 virulence [221]. The protective function against SARS-CoV-2 infection has also been assigned to NPC1. It was shown that NPC1 deficiency may be associated with obesity and insulin resistance [222].

Some cell surface-associated proteins or compounds may act as co-receptors, enhancing ACE2-dependent SARS-CoV-2 cell entry. One of such proteins is non-muscle myosin heavy chain IIA (NTG-IIA/MYH9). The C-terminal domain of the protein was able to interact with the NTD-S1 and S2 subunits of the spike protein, but SARS-CoV-2 cell entry occurred only in the presence of ACE2 [88]. The phenomenon was studied in A549, Calu-3, and H1299 cells. Although the association of the protein with diabetes is low, it has been postulated that it can be responsible, along with ACE2, for the development of pulmonary COVID-19-connected symptoms.

There are several other co-receptors of ACE2, such as heparan sulphate or scavenger receptor, class B type 1 (SR-B1), but none of them is diabetes inducible.

More focused studies are necessary to elucidate the role of the receptors of SARS-CoV-2 in the development of the COVID-19 symptom severity observed in diabetic patients. Data concerning their expression profiles and cell surface abundance in epithelial respiratory cells under hyperglycaemic conditions are crucial.

## 4. SARS-CoV-2 Spike Protein Cleavage: Diabetes-Related Implications

Proteases involved in the maturation and processing of spike protein have a significant impact on SARS-CoV-2 cell entry. While cathepsin L is downregulated in diabetes, and furin has been shown to be both up- and downregulated in serum [29,30,31,33], expression of the TMPRSS2 in cardiomyocytes increases in diabetes [25]. Studies of enterocytes revealed a relationship between a higher copy number of the SARS-CoV-2 genome in cells and a higher synthesis rate of TMPRSS2 [107]. The authors suggested even greater importance of TMPRSS2 over ACE2 during SARS-CoV-2 infection and that ACE2 can be only one of the receptors mediating SARS-CoV-2 infection since there was no correlation between viral RNA copy number and ACE2 expression in cells. On the other hand, a report by Baristaite and Gurwitz revealed that 25 mM D-galactose, applied in order to induce a diabetes-like phenotype, can stimulate mRNA synthesis of *TMPRSS2*, *ACE2,* and *FURIN* in A549 human lung epithelial cancer cells [223], which may suggest their participation in the development of COVID-19-related symptoms in the respiratory system of diabetic patients. It may also raise concerns about the impact of high milk consumption. Oral administration of 50 g of lactose increased the plasma galactose concentrations to 0.12 mmol/L, whereas 25 g of galactose increased it to 1.04 mmol/L [224]. Moreover, an increase in milk consumption reduces the risk of COVID-19 (OR = 0.9 for 98 g/day and 0.7 for 242 g/day) [225].

It seems that in spike protein cleavage, other proteases can also be implicated. Studies by Jocher et al. showed that inhibition of ADAM10 and ADAM17 activities, as well as knockdown of the two metalloproteases, reduced viral RNA levels by 70% in the tested cells [226]. ADAM17 was the one with greater influence. Moreover, at least one of the proteases may be engaged in the formation of syncytia, as observed in the COVID-19 course. Its formation may be caused by the pneumocytes expressing ACE2 in the presence of the S protein [227], but the direct functions in the process were assigned to TMEM106B since the protein is required to promote the post-endocytic stage of infection [71]. Application of ADAM10 and ADAM17 inhibitors and knockout of *ADAM10* revealed that the cell fusion observed in the pulmonary tissue may also be triggered by ADAM10 protease [226]. At least ADAM17 abundance can be induced by diabetes-associated events. Increased expression of the gene in the liver and adipose tissue of mice that were fed a diet containing high fat dosage was observed [228]. ADAM17 (TACE-TNF-α converting enzyme) is the enzyme responsible for the conversion of TNF-α, and its high expression is correlated with insulin resistance development [229]. Moreover, elevated expression of the enzyme was observed in the cells of the respiratory tract, and it was observed that its overexpression could contribute to sub-epithelial fibrosis [230].

## 5. Soluble Receptors in SARS-CoV-2 Infection

Another aspect of SARS-CoV-2 infectivity is associated with the soluble form of receptors, which appear in the blood under certain circumstances. Earlier reports have suggested that, upon SARS-CoV spike protein interaction, the abundance of ACE2 on the cell surface decreases [231]. The process, called shedding, was associated with TNF-α-converting enzyme (ADAM17) [232].

Expression analyses of ACE2 revealed that diabetes mellitus increases the concentration of the mature protein on the surface of cardiomyocytes [25] and in human kidney organoids [110]. These observations may indicate an increase in SARS-CoV-2 virulence due to induced expression of its receptor, at least in these tissues.

The relationship between soluble ACE2 (sACE2) blood concentration and diabetes is much more complicated. A higher concentration of sACE2 was observed in diabetic and obese patients [233]. Lu et al. also observed a higher serum level of ACE2, accompanied by increased angiotensin 1–7 (Ang 1–7) concentrations, but only in pre-diabetic and diabetic patients shortly after the onset of disease [234]. After 5 years, sACE2 and Ang 1–7 concentrations began to decrease, with a concomitant increase in serum ACE and Ang II levels. Decreased sACE2 levels in diabetic patients were also reported [235]. There was no such tendency in CRP and IL-6 concentrations; a constant increase was observed [234].

A similar phenomenon concerns serum ACE2 in the severe COVID-19 course. Most of the reports revealed increased COVID-19 severity dependent on sACE2 blood abundance [236]. Increased mortality among patients with high serum ACE2 was also reported [237]. However, serum concentrations of the protein in the COVID-19 course may vary. Shevchuk et al. showed that sACE2 serum level is far more elevated among home-quarantined patients with mild symptoms than in those hospitalized. The sACE2 amount increased slightly only when the disease reached its critical phase, but even then, the concentration was lower than in the control group [238]. Similar findings were reported earlier by Maza et al. [239]. Moreover, Maza et al. revealed that sACE2 serum levels were associated with the types of symptoms occurring during SARS-CoV-2 infection. Higher concentrations accompanied cutaneous symptoms, lower concentrations were assigned to gastrointestinal and upper respiratory-related symptoms, whereas pneumonia was correlated with very low sACE2 concentrations, adversely to anti-S IgG occurrence. Shevchuk et al. reported the patterns of time-dependent changes, which showed a decrease in serum ACE2 level in patients with mild symptoms but over a 30% increase in the group with severe COVID-19. Simultaneously, the authors provided information about elevated sACE2 levels in diabetic patients within all designated groups. It was concluded that loss of membrane ACE2 should be a premise of reduced viral infectivity [238]. The same conclusion has also been suggested for some other receptor candidates, including ASGR1 [61], DPP4/CD26 [240], and TfR [23]. Unless the phenomenon has been elucidated for ASGR1, DPP4/CD26, and TfR to date, Yeung et al. found that even soluble ACE2 is able to mediate SARS-CoV-2 infection through angiotensin II receptor type 1 (AT1) or vasopressin receptor (AVPR1B) in triple complex with vasopressin via endocytosis [241].

## 6. Other Factors

**Figure 4:**
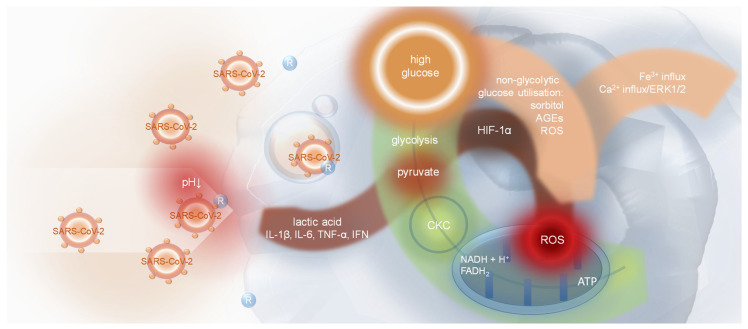
Hypoxia-mediated acidification of the extracellular space enhancing SARS-CoV-2 cell entry. ROS—reactive oxygen species; CKC—citric acid cycle; ERK1/2—extracellular signal-regulated kinases 1 and 2, associated with cell senescence; AGEs—advanced glycation end products. The arrow corresponds to the change in pH in the extracellular space.

The latest reports bring further insight into the mechanism of SARS-CoV-2–host cell infection. Kreutzberger et al. revealed that a low pH is required for the process to occur [242]. The phenomenon was reported earlier [243]. The application of high-resolution 3D live cell imaging and quantitative assays of virus infectivity distinguished three different routes of productive entry. Two of them are assumed to be related to the uptake of virus particles bound to ACE2 via endocytosis: TMPRSS2 processed or not modified, with cleavage occurring due to cathepsin activities. The third, of minor importance, is related to fusion occurring directly at the cell surface. For effective host cell infection, the virus requires a pH at or below 6.8, regardless of the route [242]. A decreased extracellular pH (7.1 or below, especially in peripheral tissues) can be observed in several pathological states. Local extracellular acidosis appears in inflammation sites, usually due to responses to pathogens [244], but also in autoimmune and allergic diseases [245]. The presence of decreased pH in the extracellular space may be explained by two distinct mechanisms. One of the potential mechanisms could be the accumulation of lactate under hypoxic conditions. The process is not obligatorily associated with cells of the immune system and can be observed in the pathology of T2D [246], for example, in renal proximal tubular epithelial cells. These cells isolated from diabetic patients exhibited intensified glycolysis at the expense of altered mitochondrial respiration [110]. Earlier studies indicated the involvement of hypoxia-inducible factor 1 (HIF-1) in the process [247]. HIF-1 is also able to trigger lactate dehydrogenase A (LDHA) overexpression [248]. “Aerobic glycolysis” results in lactate synthesis even in the presence of an adequate amount of oxygen, which is then excluded from cells by monocarboxylate transporters. Inhibition of “aerobic glycolysis” revealed reduced SARS-CoV-2 infection susceptibility [110]. Studies on the effect of hyperglycaemia on monocytes infected with SARS-CoV-2 revealed the favourable impact of high glucose on the virus replication rate. The process was completely inhibited by 2-deoxyglucose and galactose, which was specific to SARS-CoV-2 since the replication rate of other tested viruses was not affected. Virion replication was also suppressed by the inhibition of lactate dehydrogenase. Overexpression of *HIF-1α*, a key inducer of both the Warburg effect and increased cytokine synthesis in monocytes (IL-1β, IL-6, TNF-α, and IFN-α/β/γ), was the basis of the occurring changes. The process was accompanied by increased mitochondrial reactive oxygen species (ROS) generation, inducing viral genome amplification. MitoQ inhibited the effect. This led to reduced T cell proliferation and lung epithelial cell death in the presence of infected monocytes or cultured in virus-inactivated media obtained from cultures of infected monocytes [249]. It suggests that lactate may induce SARS-CoV-2 internalisation in a pH-dependent manner since viral replication seems to be related to the induction of aerobic glycolysis. The basic assumptions of the process are presented in Figure 4.

The second potential mechanism of acidification may be associated with the direct action of macrophages. It was shown that, in specific circumstances, macrophages are able to acidify the local extracellular matrix. Such a phenomenon was observed in atherogenesis. One of the key steps of cholesteryl ester hydrolysis was the release of lysosomal content into the extracellular space upon contact with LDL aggregates, resulting in increased levels of free cholesterol, which was then taken up [250]. The large numbers of ATM cells involved in white adipose tissue remodelling events suggest the possibility of the functioning of such a process in adipose tissue. ATM migration is correlated with adipose tissue cell death [251]. It influences not only the number of macrophages present in the tissue but also the accumulation of lipids in the ATM cytoplasm. A connection between lipolysis in the adipose tissue of obese individuals and intensified lysosomal biogenesis in ATMs was observed, suggesting that a similar previously described event characteristic of atherogenesis may also be applicable in the case of obesity.

The described phenomena seem to be important since the spike protein–receptor (ACE2) interaction may be acid-base. Studies by Hristova and Zhivkov revealed the difference in pI between ACE2 and the trimeric form of the S protein. The pI values were estimated at 5.4 and 7.3, respectively. Moreover, it was shown that spike protein mutations influence both the contagiousness and pathogenicity of the SARS-CoV-2 omicron variant via the strength of pH-dependent ACE2 binding, which is weaker at pH 7 but stronger at lower pH [252]. A similar nature of the interactions was proposed for the ACE-2 co-receptors heparan sulphate and sialic acid derivatives [82].

The pH value of the respiratory track differs from that observed in blood plasma and in the extracellular space. The pH of the nasal mucosa was established at 5.3–7.0, slightly higher in men than in women [253], and was not dependent on age. The pH of the extravascular space of the lung is 6.69 ± 0.07 [254]. It may facilitate the entry of the virus into the cell. The pH of the nasal mucosa was increased in diabetic patients [255], but pneumonia may generate hypoxia and lactate synthesis in close proximity to pneumocytes within endothelial cells [256]. Further studies are needed to elucidate the pH influence on SARS-CoV-2 virulence and its connection to diabetes.

## 7. Diabetes and Long COVID-19

Studies of long COVID-19 revealed that diabetes type 2 is not a risk factor for associated disorder development, with OR = 1.06. Similar observations considered any of the most common symptoms [257]. Higher risk is associated with obesity (BMI > 30 kg/m^2^, OR = 1.12). [258]. On the other hand, among patients that developed long COVID, diabetes type 2 is one of the emerging disorders. The risk of T2D occurrence after the acute phase of SARS-CoV-2 infection was 60% higher than in the non-diabetic population and higher than in the group of patients who endured other types of respiratory infections [259].

## 8. Conclusions

The influence of diabetes mellitus type 2 on severe COVID-19 induction is complex and concerns many aspects of glucose metabolism. This article does not concern many of the topics that should be discussed to provide comprehensive insights into the existing connections and their impact on viral infections. However, the aspect of the functioning of SARS-CoV-2 receptors in diabetes, which can contribute to the worsening of COVID-19 outcomes, seems to be of particular importance since it directly affects the ability of the virus to multiply. Among the proteins that show the ability to bind SARS-CoV-2 spike proteins, some display increased representation in diabetes, whereas the expression of others is decreased. The synthesis rate may be tissue dependent, and many unrecognised factors may affect the binding strength as well as the abundance of the protein at the cell surface. Moreover, the results concern only some aspects of the functioning of SARS-CoV-2 receptors in diabetes. However, several proteins seem to be important since diabetes-related disorders may affect their membrane abundance. Comprehensive studies are needed concerning the receptors’ expression profiles, regulation of the membrane incorporation process, and the influence of diabetes-associated conditions on SARS-CoV-2 spike protein binding and virus internalisation in order to understand the impact of the disease on the worsening of COVID-19 outcomes. It may also be beneficial for understanding the functioning of viruses in general. Studies on MERS-CoV or SARS-CoV allowed for the development of schemes, the application of which facilitated research on the new coronavirus. However, not sufficiently. Much attention should now be paid to the explanation of molecular mechanisms that are responsible for the deterioration of the outcomes of patients with infectious diseases. Learning how to prevent them may be beneficial for the future.

## Figures and Tables

**Table 1:** Potential receptors of SARS-CoV-2, essential proteases, and their regulation in diabetes.

Factor	Proposed Mechanism	Expression In Diabetes	Cell Type/Tissue *
ACE2	via RBD binding [5,15,24]	increased in diabetes mellitus [25]	cardiomyocytes
		decreased in diabetes and obesity [26]	adipose tissue
TMPRSS2 **	via cleavage of S protein at S2’ site [15,27]	increased in diabetes mellitus [25]	cardiomyocytes
Furin **	via cleavage of S proprotein at S1/S2 site [27]	negatively associated with fasting glucose [28]	serum
		positively associated with fasting glucose, insulin, and BMI [29,30,31]	plasma/serum
cathepsin L **	via cleavage S protein at CS-1 and CS-2 sites [32]	decreased in diabetes mellitus [33]	endothelial progenitor cells
		decreased in diabetes mellitus [34]	mouse models
CD147	not specified [35], via RBD binding [36], via regulation of abundance of ACE2 native protein [37], via CyPA mediated S binding [38]	increased due to high glucose [39]	adipose tissue
		increased in obesity and diabetes mellitus [39]	blood plasma
GRP78	via S binding [40]	increased at the cell surface due to high glucose [41]	rat glomerular mesangial cells
		increased at the cell surface due to high glucose [42]	HUVECs
DPP4/CD26	via S binding in silico [43]	marker of visceral obesity and insulin resistance [44]	human blood plasma
TfR	via intact S binding [23]	increased in diabetes [45]	blood plasma
		insulin inducible membrane association [46]	rat adipocytes
		streptozotocin-induced diabetes [47]	rat kidney
integrin α_5_β_1_	via RGD motif of spike protein [48,49]	increased due to high glucose [50,51]	HUVECs;
			mouse mesangial and glomerular endothelial cells
integrin α_v_β_3_	via RGD motif of spike protein [52,53]	increased in diabetic nephropathy [54]	epithelial cells
		increased in diabetes [55]	rat’s endometrium
CD4	via S/RBD binding [56]	increased number of CD4^+^ T cells in T2D-related visceral obesity [57]	lymphocytes
mGluR2	via virion internalisation, S binding ability [58]	increased in streptozotocin-induced diabetes [59]	rat hippocampal neurons
		increased in neonates of rats with streptozotocin-induced diabetes [60]	rat lateral geniculate body
ASGR1	via RBD and NTD binding [61,62]	decreased in diabetes [63]	peripheral blood
			mononuclear cells
KREMEN1	via RBD and NTD binding [62]	NA	
AXL	via NTD binding [64], via apoptotic mimicry [65]	decreased due to high glucose [66]	microvascular endothelial cells
CD209L/L-SIGN,	via S binding, ACE2 co-receptor [67]	NA	
CD209/DC-SIGN			
FcγRIII (CD16)	via antibody binding [68]	monocyte number decreased in diabetes; CD16^+^ monocytes decreased in diabetes with complications [69]	monocytes
FcγRI (CD64)		CD64 expression was not altered by diabetes [70]	macrophages
TMEM106B	via RBD binding, syncythium formation [71]	NA	
NPC1	via nucleoprotein interaction [72]	chemical and genetic inhibitors impair insulin signalling [73]	adipocytes
NRP1	via S binding in co-expression with ACE2 and TMPRSS2 [74,75]	increased in hypoglycaemia [76]	blood plasma
		decreased due to glycated BSA [77]	mouse podocytes
NRP2	unspecified, probable ACE2 co-receptor [78]	highly inducible in proinflammatory state [78]	fibroblasts
vimentin	via RBD binding, ACE2 co-receptor [79]	secreted in response to oxLDL [80]	subcutaneous adipocytes
		increased due to high glucose [81]	vascular endothelial cells
sialic acid (monosialylated gangliosides)	via RBD binding/ACE2 co-receptor [82]	increased in diabetes and diabetic nephropathy [83]	human serum
		increased in senescence [84]	endothelial cells
heparan sulphate	via S binding, ACE2 co-receptor [85]	decreased in diabetes mellitus [86]	mesangial and glomerular visceral epithelial cells
		reduction promotes mice β-cell failure [87]	mice β-cells
NTG-IIA	via S binding to C-terminal domain, ACE2 co-receptor [88]	increased activity due to exposure to macroalbuminuric sera of T1D patients [89]	podocytes,
			rat glomeruli
SR-B1	via HDL-mediated S binding, in co-expression with ACE2 [90]	decreased in hyperglycaemia [91]	HepG2

NA—data not available; * applies to human cells/tissues, unless stated otherwise; ** proteases essential for ACE2—SARS-CoV-2 interactions; Abbreviations: ACE2—angiotensin converting enzyme 2; RBD—SARS-CoV-2 receptor binding domain; TMPRSS2—transmembrane serine protease 2; S—SARS-CoV-2 spike protein; BMI—body mass index; CD147—basigin; CyPA—cyclophilin A; GRP78—glucose regulated protein 78; HUVECs—human umbilical vein endothelial cells; DPP4/CD26—dipeptidyl peptidase 4; TfR—transferrin receptor; RGD—Arg-Gly-Asp motif; CD4—cluster of differentation 4; mGluR2—metabotropic glutamate receptor 2; ASGR1—asialoglycoprotein receptor 1; NTD—N-terminal domain of the SARS-CoV-2 nucleprotein; KREMEN—Kringle Containing Transmembrane Protein 1; AXL—tyrosine-protein kinase receptor UFO; CD209L/L-SIGN, CD209/DC-SIGN—C-type lectins; TMEM106B—transmembrane protein 106B; FcγRIII/I (CD16/64)—IgG FC receptors III and I, respectively; NPC1—Niemann–Pick disease, type C1; NRP1/2—neuropilin 1/2; BSA—bovine serum albumin; oxLDL—oxidized low-density lipoprotein; NTG-IIA—non-muscle myosin heavy chain IIA (MYH9); T1D—diabetes mellitus type 1; SR-B1—scavenger receptor class B member/type 1; HepG2—epithelial-like cell line, hepatocellular carcinoma.

## Supplementary Materials

The supporting information can be downloaded at: https://www.mdpi.com/article/10.3390/ijms25179635/s1.
