# Supplementary material for: The Functions of SARS-CoV-2 Receptors in Diabetes-Related Severe COVID-19
Source: Int J Mol Sci. 2024 Sep 5;25(17):9635. doi: 10.3390/ijms25179635 (PMC11394807; doi:10.3390/ijms25179635)
Supplement: Supplementary file 1 [file ijms-25-09635-s001.zip › ijms-3163027-supplementary.pdf]

Supplementary materials

The functions of SARS-CoV-2 receptors in diabetes-related severe COVID-19

Adam Drzymała [1,*]

[1] Department of Clinical Biochemistry and Laboratory Diagnostics, Institute of Medical Sciences, University of Opole, Oleska 48, 45-052 Opole, Poland
* Correspondence: adam.drzymala@uni.opole.pl

List of genes from the Human Protein Atlas with URLs:

In **main text**; in the order of appearance:
AXL – https://www.proteinatlas.org/ENSG00000167601-AXL/tissue;
NRP1 – https://www.proteinatlas.org/ENSG00000099250-NRP1/tissue;
TfR – https://www.proteinatlas.org/ENSG00000072274-TFRC/tissue;
GRP78 – https://www.proteinatlas.org/ENSG00000044574-HSPA5/tissue;
mGluR2 – https://www.proteinatlas.org/ENSG00000164082-GRM2/tissue,

available from Human Protein Atlas [92], v23.0.proteinatlas.org

In the **Figure 1**; in the order of appearance:
ACE2 – www.proteinatlas.org/ENSG00000130234-ACE2;
DPP4 – www.proteinatlas.org/ENSG00000197635-DPP4;
CD4 – www.proteinatlas.org/ENSG00000010610-CD4;
TfR – www.proteinatlas.org/ENSG00000072274-TFRC;
CD147 – www.proteinatlas.org/ENSG00000172270-BSG;
ASGR1 – www.proteinatlas.org/ENSG00000141505-ASGR1;
AXL – www.proteinatlas.org/ENSG00000167601-AXL;
TMEM106B – www.proteinatlas.org/ENSG00000106460-TMEM106B;
NRP1 – www.proteinatlas.org/ENSG00000099250-NRP1;
NRP2 – www.proteinatlas.org/ENSG00000118257-NRP2;
GRP78 – www.proteinatlas.org/ENSG00000044574-HSPA5,
available from Human Protein Atlas [92], v23.0.proteinatlas.org
